# Supplementary material for: Luminescent hyperbolic metasurfaces
Source: Nat Commun. 2017 Jan 9;8:13793. doi: 10.1038/ncomms13793 (PMC5473634; doi:10.1038/ncomms13793)
Supplement: Supplementary Information — Supplementary Figures, Supplementary Notes and Supplementary References. [file ncomms13793-s1.pdf]

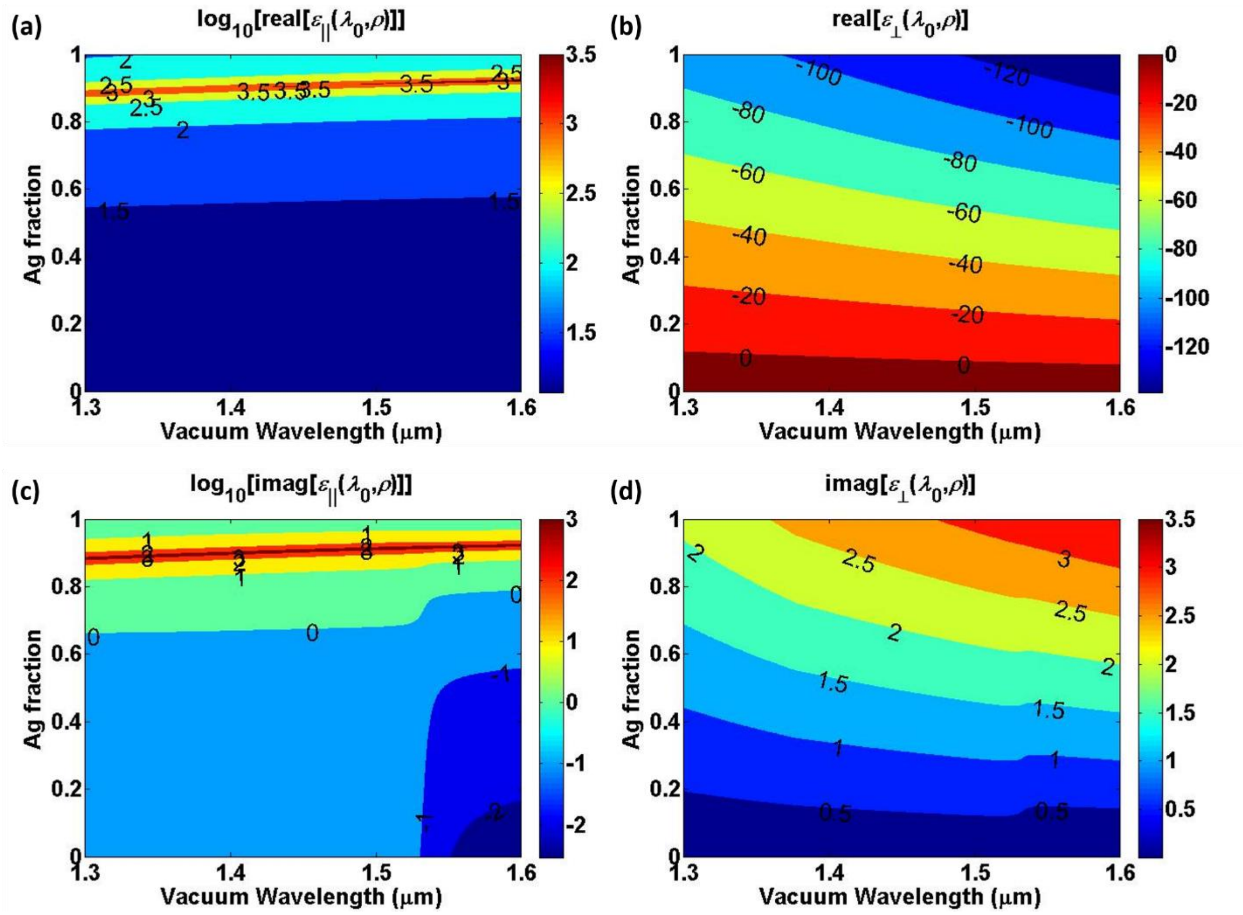

Supplementary Figure 1. Hyperbolic dispersion in the effective medium limit. (a,b) Real parts of the effective permittivity elements (a) parallel and (b) normal to the metacrystal Bloch vector, $\mathbf{K}_B$. Hyperbolic dispersion occurs for $\varepsilon_{||}'\varepsilon_{\perp}' < 0$, which is satisfied over the entire Ag fraction-wavelength parameter space except for Ag fractions below ~0.1. (c,d) Imaginary parts of the effective permittivity elements (c) parallel and (d) normal to $\mathbf{K}_B$, for N=1x10^{16}cm^{-3}. Dissipation generally increases with metal fraction due to Ohmic losses in Ag and increases abruptly around λ_0=1.55μm due to absorption at the band-edge of the InGaAsP MQW. Note that (a,c) are in log-scale due to the resonance at ρ≈0.9.

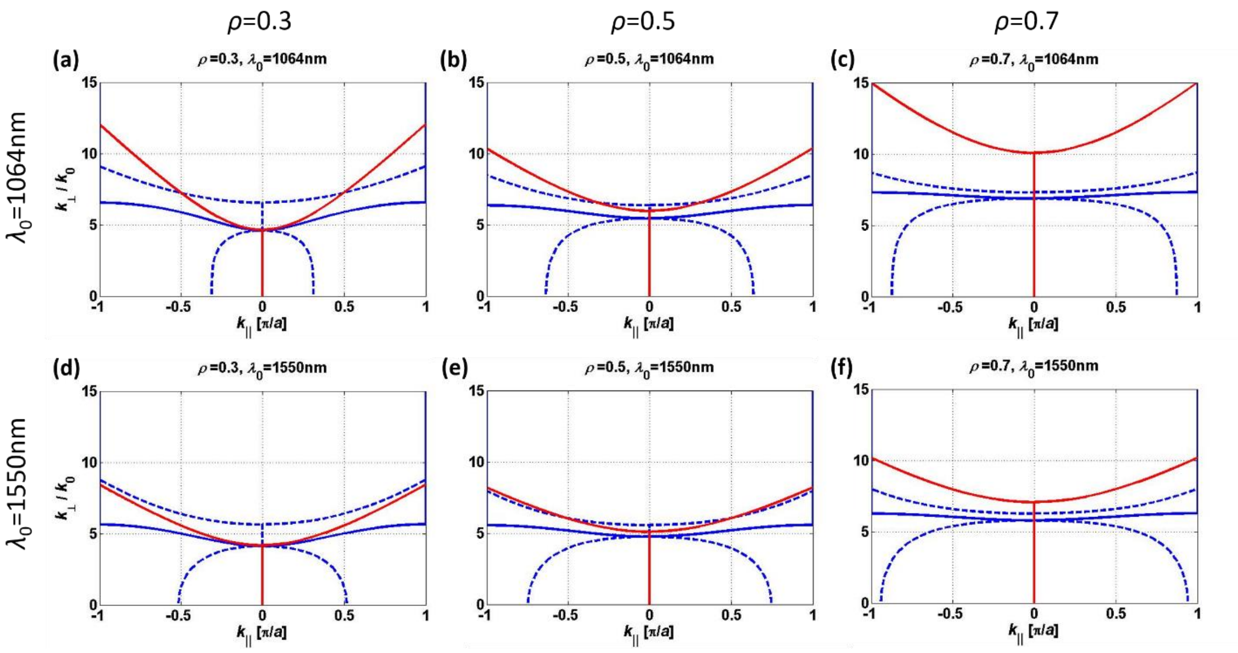

Supplementary Figure 2. Hyperbolic dispersion in Ag/InGaAsP multilayer materials illustrated by wave-vector diagrams. Solid (dashed) blue curves are the real (imaginary) part of the solution to Eq.(1), while red curve is solution to Eq. (6). (a-c) Solutions for pump wavelength of λ_0=1064 nm and Ag fractions of (a) ρ=0.3, (b) ρ=0.5, and (c) ρ=0.7. (d-f) Solutions for emission wavelength of λ_0=1550 nm and Ag fractions of (d) ρ=0.3, (e) ρ=0.5, and (f) ρ=0.7.

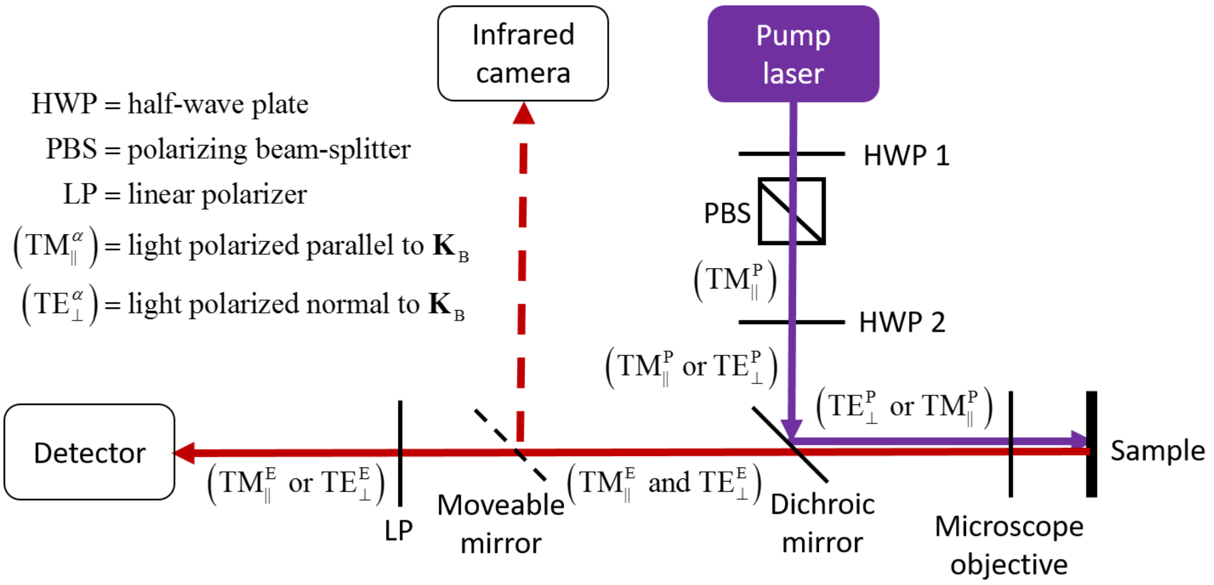

Supplementary Figure 3. Polarization-resolved micro-photoluminescence experiment. The superscripts for light polarization are α=P and α=E for pump and emission, respectively.

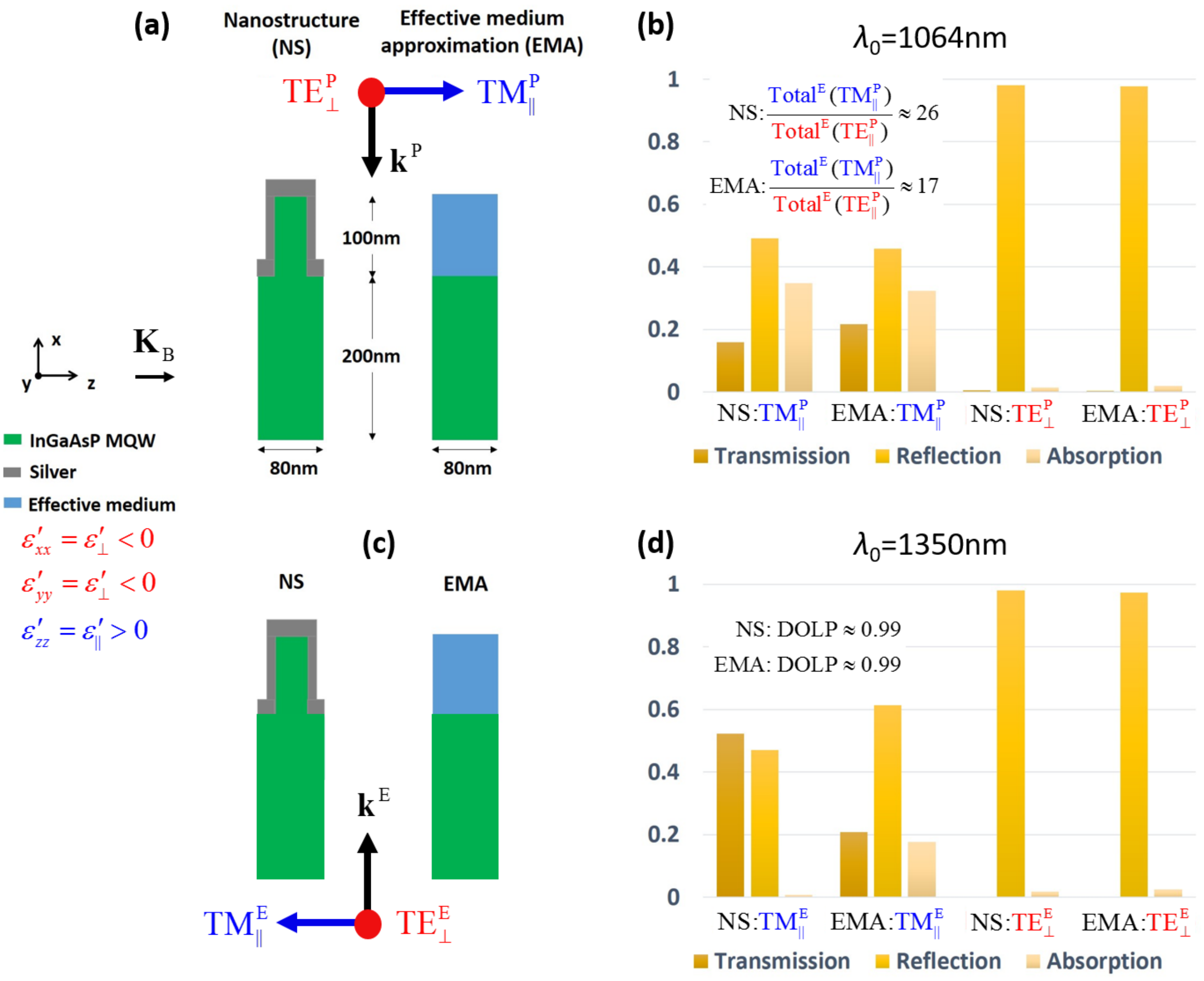

Supplementary Figure 4. Simulated polarization dependence of pump and polarization-resolved emission. (a) Schematic of pump simulation for actual structure and effective medium approximation (EMA). (b) Transmission, reflection, and absorption of pump when electric field is polarized parallel ($TM_{\parallel}^{P}$) and normal ($TE_{\perp}^{P}$) to metacrystal Bloch vector, $\mathbf{K}_B$ (z-direction). Strong pump polarization anisotropy (PA) is observed in the simulation, consistent with experimentally determined pump PA. (c) Schematic of emission simulation. (d) Transmission, reflection, and absorption of emission when electric field is polarized parallel ($TM_{\parallel}^{E}$) and normal ($TE_{\perp}^{E}$) to $\mathbf{K}_B$. Transmitted emission is highly polarized, consistent with experimentally measured PL. The simulated results in the EMA are nearly identical to results for the NS, indicative that the fabricated samples behave as hyperbolic metasurfaces.

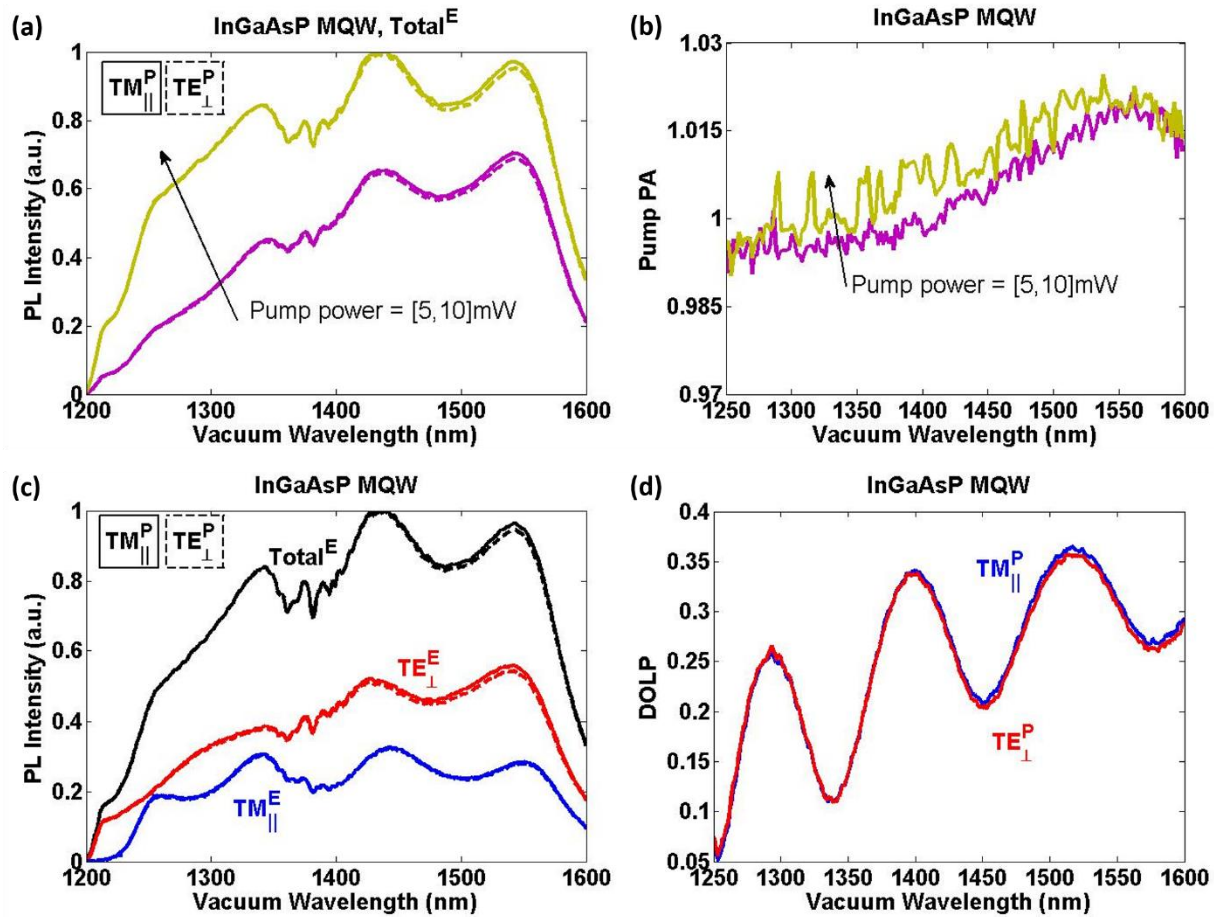

Supplementary Figure 5. Characterization of InGaAsP MQW control. (a) Total PL for parallel-polarized (solid lines) and normal-polarized (dashed lines) pump for two power levels. (b) Pump PA calculated from (a), showing negligible dependence of PL on pump polarization. (c) PL resolved into parallel and normal polarization components for both parallel-polarized (solid lines) and normal-polarized (dashed lines) pump. (d) Degree-of-linear-polarization (DOLP) of PL showing modest polarization and no dependence on pump polarization.

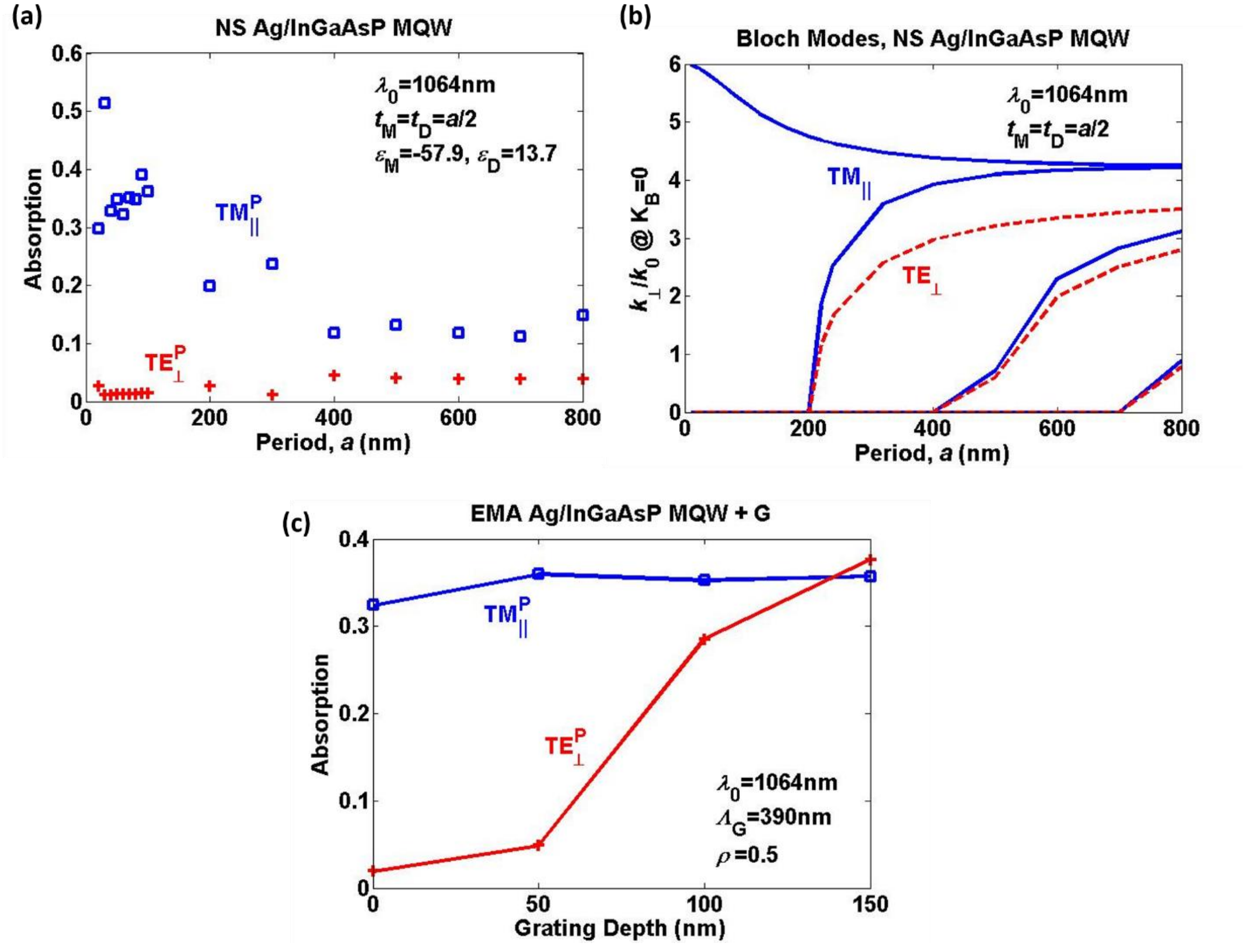

Supplementary Figure 6. Simulated pump anisotropy of LuHMS. (a) Simulated absorption of pump polarized parallel and normal to $\mathbf{K}_B$ as a function of the Ag/InGaAsP period length, with layer thicknesses fixed at $t_M=t_D=a/2$. Polarization anisotropy is significantly greater than a factor of 2 only for periods less than 200 nm. (b) Geometric dispersion of Bloch modes. Magnitude of wave-vector component normal to $\mathbf{K}_B$ as a function of period length, for $\mathbf{K}_B=0$, $\lambda_0=1064$ nm, and $t_M=t_D=a/2$. (c) Simulated absorption of parallel and normal-polarized pump as a function of grating depth for 390 nm grating period using EMA with $\rho=0.5$. Polarization anisotropy is reduced significantly in the presence of a grating.

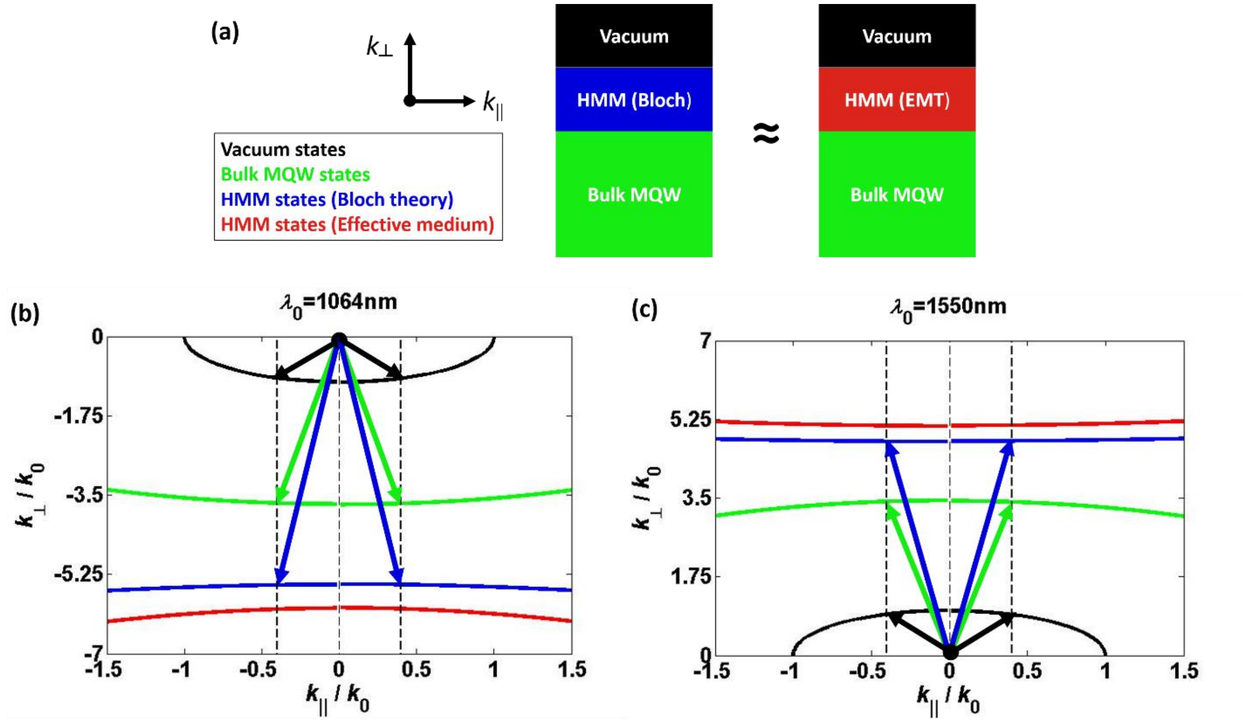

Supplementary Figure 7. Description of coupling between high-*k* HMM states and vacuum states without grating coupler. (a) Schematic of LuHMS indicating wave-vector components parallel and normal to optical axis. Wave-vector diagram for LuHMS at (b) λ_0=1064 nm and (c) λ_0=1550 nm, showing HMM states predicted by EMT and Bloch Theory, along with states in the bulk MQW and vacuum. The angular bandwidth in vacuum is determined by the experimental excitation and detection half-angle of 24° from normal incidence.

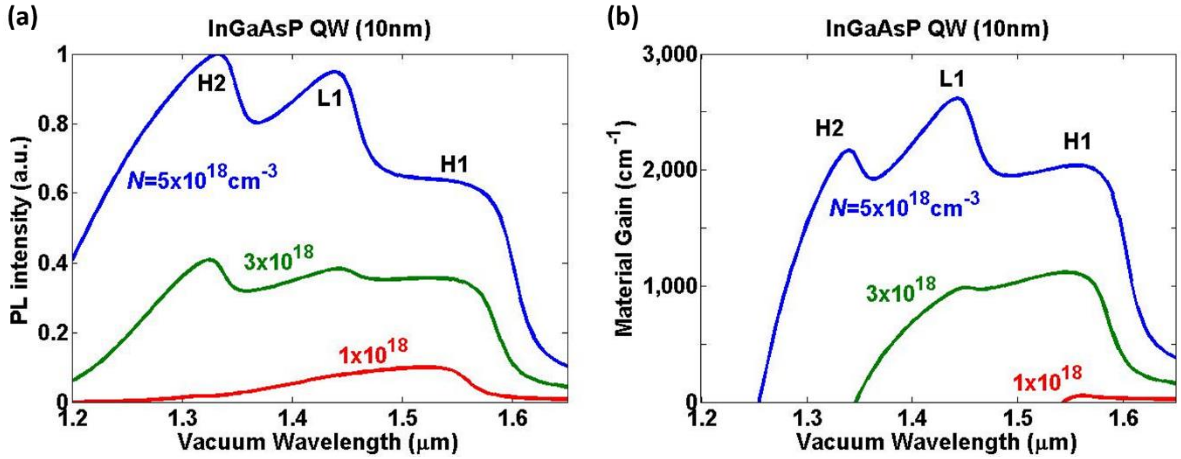

Supplementary Figure 8. Theoretical PL and material gain spectra of InGaAsP QW. Calculated (a) spontaneous emission and (b) gain spectra with carrier density, *N*, as a parameter. Transitions between the conduction band and (H1) first heavy-hole, (L1) first light-hole, and (H2) second heavy-hole subbands are noted.

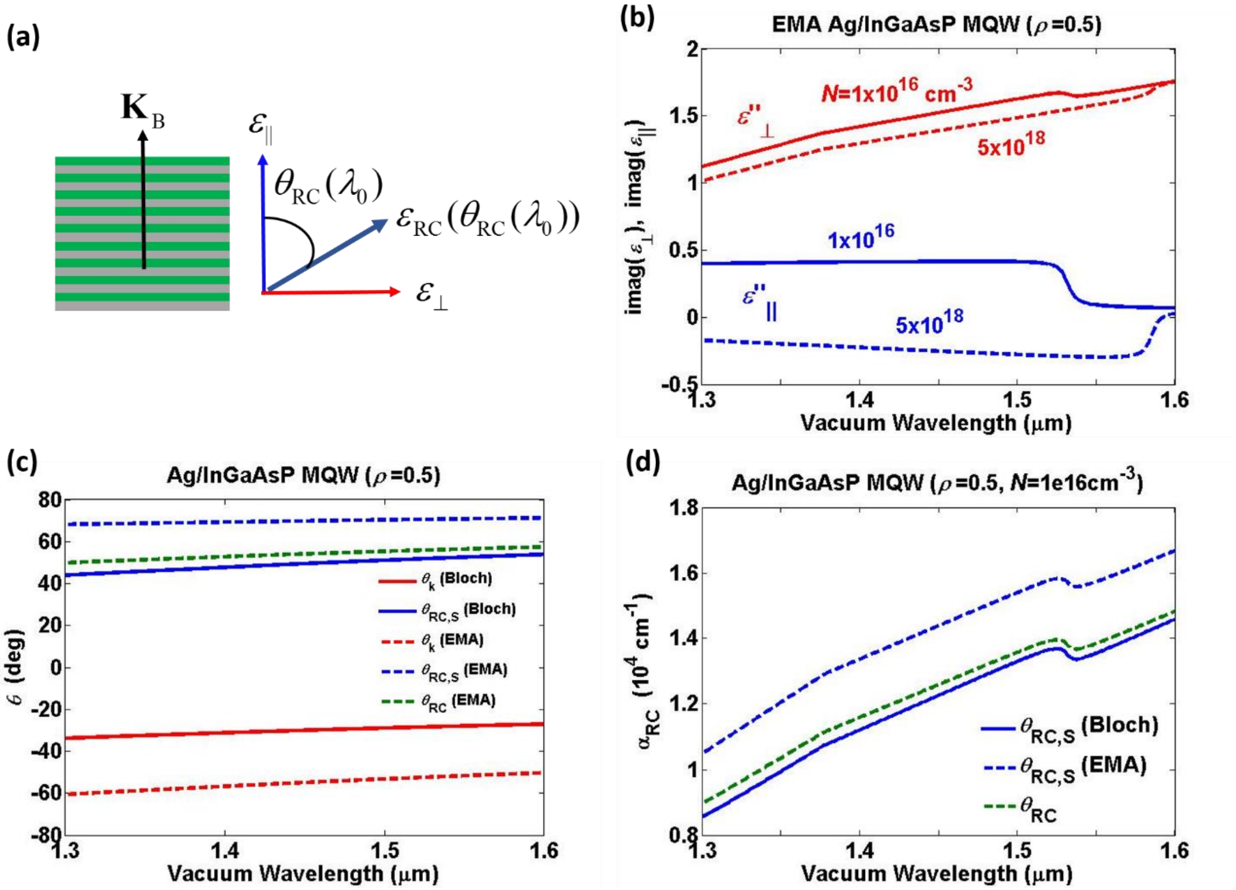

Supplementary Figure 9. Wavelength dependence of principal direction of energy propagation and effective attenuation. (a) Schematic top-down view of multilayer with resonance cone angle defined with respect to metacrystal Bloch vector, $\mathbf{K}_B$. (b) Dispersion of imaginary parts of effective permittivity elements parallel and normal to $\mathbf{K}_B$, calculated by Eqs. (7)-(8) for Ag fraction of 0.5. (c) Wavelength dependence of Poynting and wave vectors calculated according to Eqs. (9)-(11). (d) Dispersion of attenuation in direction of Poynting vector calculated by Eq. (12)-(13). Effective attenuation increases monotonically with wavelength because the resonance cone angle increases with wavelength.

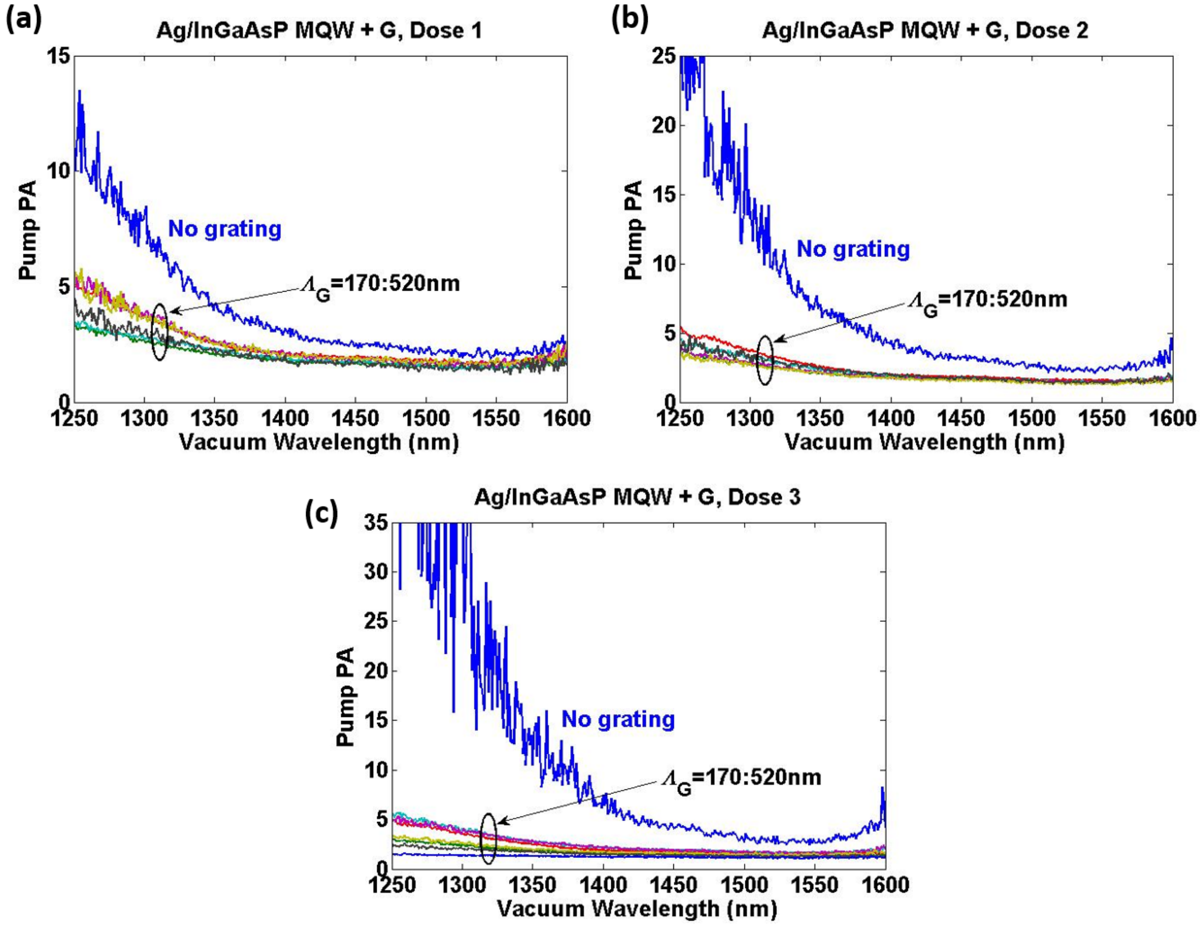

Supplementary Figure 10. Measured pump PA in LuHMS with gratings and tolerance of PA to fabrication variability. Reduction of pump PA of PL in LuHMS samples by incorporation of grating couplers, showing consistency in reduction of PA with gratings of periods ranging from 170nm to 520nm. (a-c) Three samples exposed to increasingly intense electron beam doses.

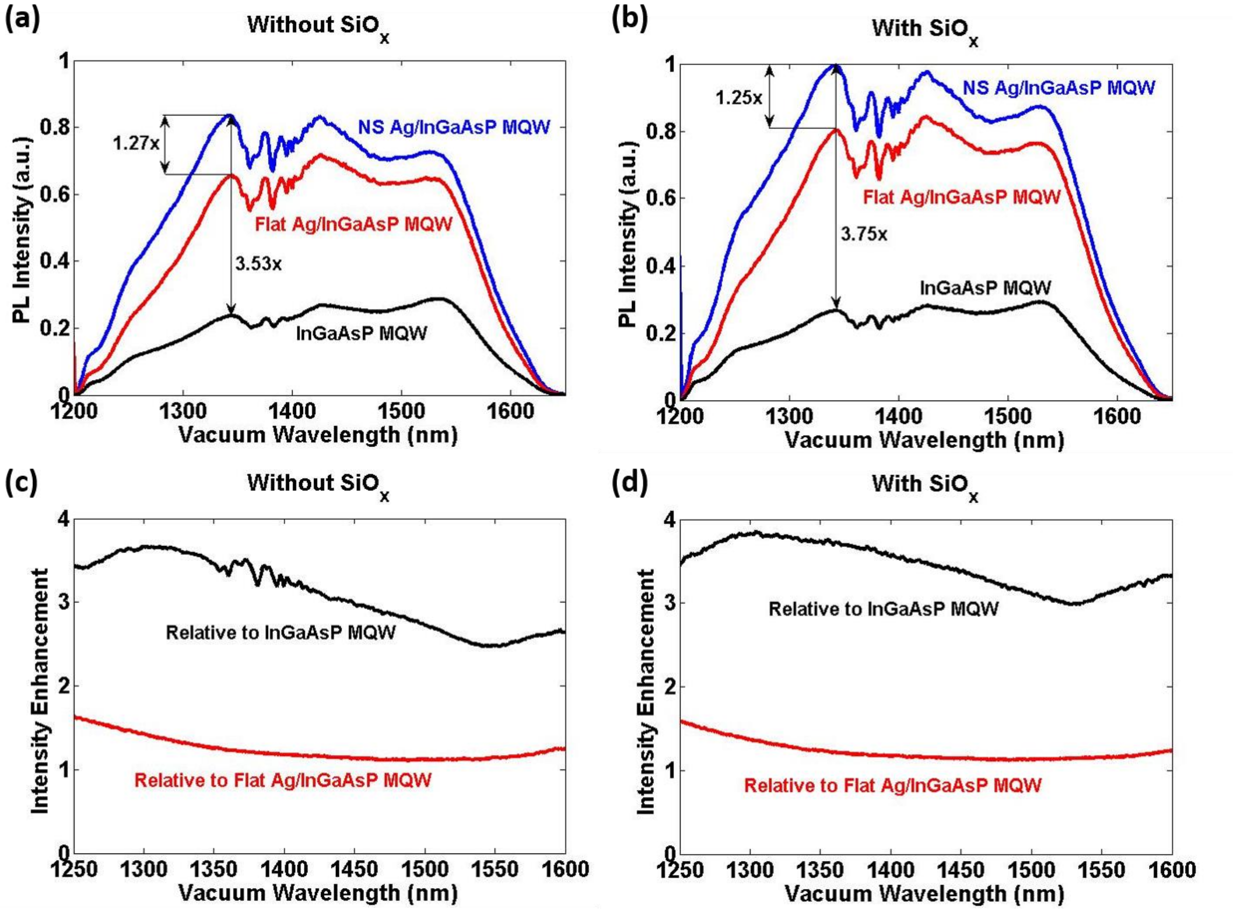

Supplementary Figure 11. Enhancement of PL spectra. (a,b) PL spectra and (c,d) intensity enhancement spectra in LuHMS (a,c) without and (b,d) with SiO_x insulation layer, under reverse excitation and fixed average pump power of 5 mW. Relative to InGaAsP MQW and a single, flat Ag/InGaAsP MQW interface, the nanostructured Ag/InGaAsP MQW system exhibits roughly 3.5x and 1.25x stronger PL intensities across the emission spectrum. Total PL signal of flat and nanostructured Ag/InGaAsP MQW is increased by ~1.25x in the presence of SiO_x, suggesting that quenching is reduced via insulation layer.

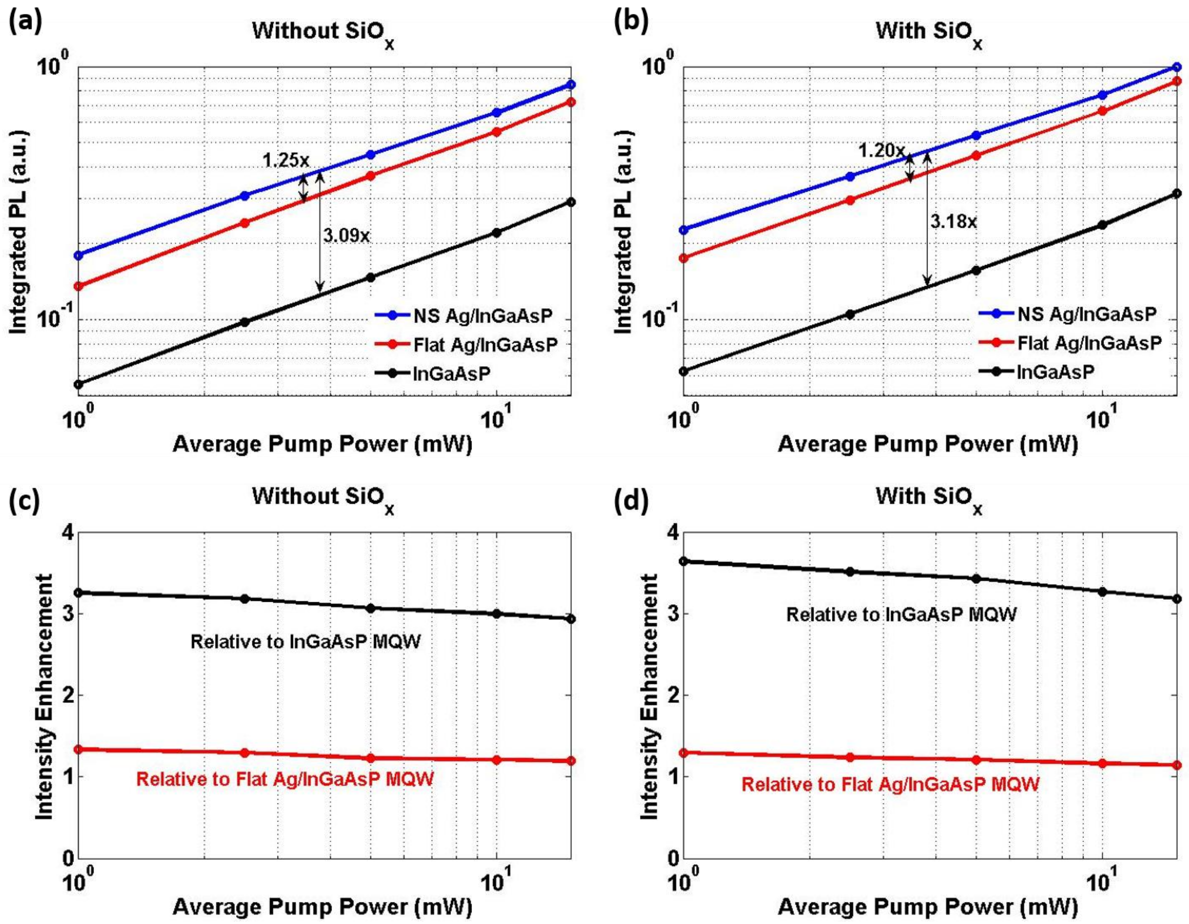

Supplementary Figure 12. Enhancement of integrated PL. (a,b) Integrated PL and (c,d) integrated intensity enhancement in LuHMS as a function of average pump power (a,c) without and (b,d) with SiO_x insulation layer under reverse excitation. Relative to InGaAsP MQW and a single, flat Ag/InGaAsP MQW interface, the nanostructured Ag/InGaAsP MQW system exhibits roughly 3.0x and 1.25x stronger PL intensities across the range of studied pumping power. Total PL signal of flat and nanostructured Ag/InGaAsP MQW is increased by ~1.25x in the presence of SiO_x, suggesting that quenching is reduced via insulation layer.

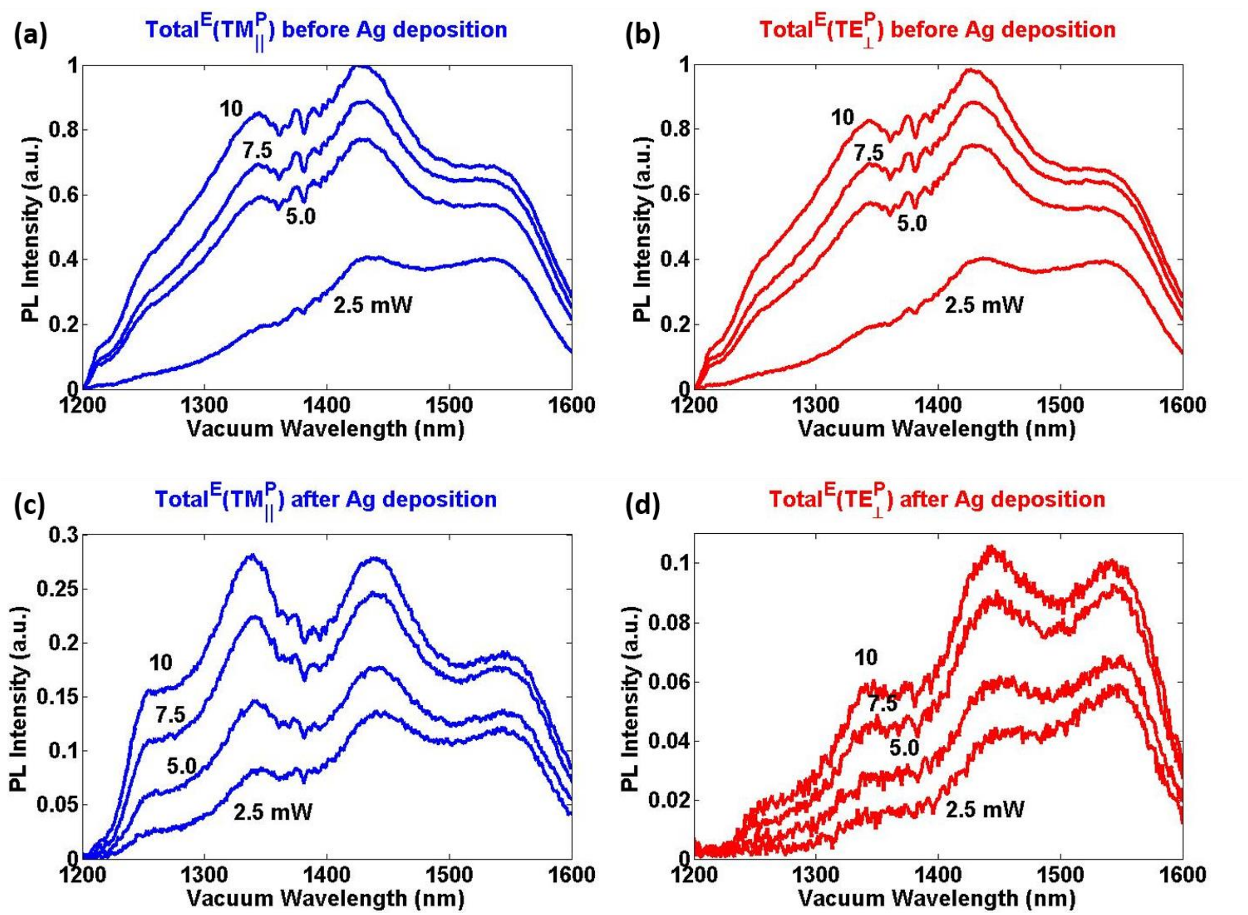

Supplementary Figure 13. Comparison of PL spectra before and after Ag deposition. Total PL spectra of nanostructured InGaAsP MQW (a,b) before and (c,d,) after Ag deposition for pump polarized (a,c) parallel and (b,d) normal to metacrystal Bloch vector, with average pump power as a parameter. Spectra are normalized to peak value before Ag deposition.

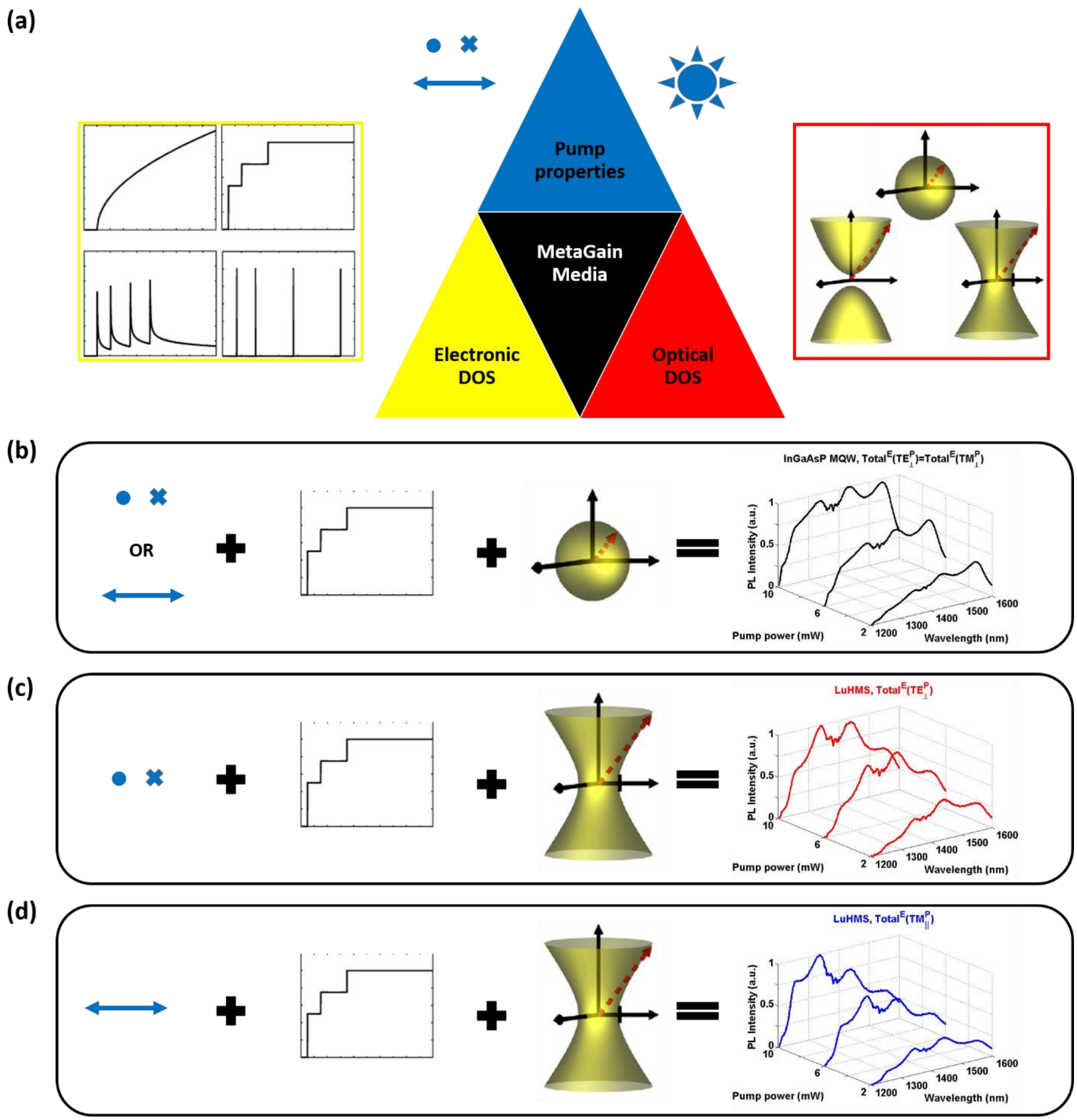

Supplementary Figure 14. Principle and realization of meta-gain media. (a) Simultaneous co-optimization of pump properties, electronic density of states (DOS) and optical DOS enables, in principle, tailor-made emission spectra with gain properties beyond those of constituent materials. Evolution of PL spectrum with power for (b) control InGaAsP MQW, (c) LuHMS with normal-polarized pump, and (d) LuHMS with parallel-polarized pump. (Electronic and optical DOS schematics were modified from[1] and[2], respectively.)

Supplementary Note 1. Description of the Ag/InGaAsP MQW system under the effective medium approximation (EMA) and Bloch's theorem.

The EMA is a powerful tool for gaining intuition about complex composite media[3]. Essentially, the EMA transforms the periodic, inhomogeneous Ag/InGaAsP system into an anisotropic, homogeneous material, with properties governed by those of the constituent materials and their respective ratio. We here show that, under both the EMA and Bloch's theorem, the Ag/InGaAsP system exhibits hyperbolic dispersion for a wide range of ratios throughout the telecommunication and near-infrared frequency range.

We first approximate our fabricated structure as a one-dimensional infinitely periodic system. Bloch's theorem may then be invoked to determine the range of transverse momentum states supported by the system, $\Delta k_\perp$. This is done by solving for the Bloch vector, $\mathbf{K}_\mathrm{B}$, of the system, given by[4]

$$\mathbf{K}_\mathrm{B} = -\frac{1}{a}\cos^{-1}\left(\frac{A+D}{2}\right) + \frac{2\pi}{a}s, \quad s = 0,1,2... \ , \tag{1}$$

where

$$A = \exp\left(ik_{\|,\mathrm{M}}t_\mathrm{M}\right)\cos\left(k_{\|,\mathrm{D}}t_\mathrm{D}\right) + \frac{1}{2}i\left(\frac{\varepsilon_\mathrm{D}k_{\|,\mathrm{M}}}{\varepsilon_\mathrm{M}k_{\|,\mathrm{D}}} + \frac{\varepsilon_\mathrm{M}k_{\|,\mathrm{D}}}{\varepsilon_\mathrm{D}k_{\|,\mathrm{M}}}\right)\sin\left(k_{\|,\mathrm{D}}t_\mathrm{D}\right) \tag{2}$$

$$D = \exp\left(-ik_{\|,\mathrm{M}}t_\mathrm{M}\right)\cos\left(k_{\|,\mathrm{D}}t_\mathrm{D}\right) - \frac{1}{2}i\left(\frac{\varepsilon_\mathrm{D}k_{\|,\mathrm{M}}}{\varepsilon_\mathrm{M}k_{\|,\mathrm{D}}} + \frac{\varepsilon_\mathrm{M}k_{\|,\mathrm{D}}}{\varepsilon_\mathrm{D}k_{\|,\mathrm{M}}}\right)\sin\left(k_{\|,\mathrm{D}}t_\mathrm{D}\right) \ . \tag{3}$$

In Eq. (1) a is the length of one period, equal to the sum of the Ag and InGaAsP layer thicknesses, t_M and t_D, respectively. In Eqs. (2)-(3) the complex, frequency dependent dielectric function of Ag, ε_M, is based on experimental data[5], whereas that of InGaAsP, ε_D, is based on a combination of experimental[6] and theoretical data[7,8]. Furthermore, ε_D also depends upon the free carrier density, N, which is controlled by external pumping. The longitudinal wave components within the Ag and InGaAsP layers, $k_{\|,\mathrm{M}}$ and $k_{\|,\mathrm{D}}$, respectively, are related to the conserved transverse component, $k_\perp$, and vacuum wavenumber, $k_0=2\pi/\lambda_0$ by

$$k_{\|,\mathrm{M}} = \sqrt{\varepsilon_\mathrm{M}k_0^2 - k_\perp^2} \tag{4}$$

$$k_{\|,\mathrm{D}} = \sqrt{\varepsilon_\mathrm{D}k_0^2 - k_\perp^2} \ . \tag{5}$$

Experimentally, we verified that $t_\mathrm{M}\approx t_\mathrm{D}\approx$40nm. However, to account for sample non-uniformities we allow the material ratio, $\rho=t_\mathrm{M}/t_\mathrm{D}$, to vary, while keeping the period fixed to a=80nm. The zeroth-order EMA of Eq. (1) is

$$k_{\mathrm{B,EMA}} = -\sqrt{\varepsilon_{\perp}\left(1-\frac{k_{\perp}^{2}}{\varepsilon_{\parallel}}\right)}\ , \tag{6}$$

where

$$\varepsilon_{\perp} = \rho\varepsilon_{\mathrm{M}} + (1-\rho)\varepsilon_{\mathrm{D}} = \varepsilon_{\perp}' + i\varepsilon_{\perp}'' \tag{7}$$

$$\varepsilon_{\parallel} = \frac{\varepsilon_{\mathrm{M}}\varepsilon_{\mathrm{D}}}{(1-\rho)\varepsilon_{\mathrm{M}} + \rho\varepsilon_{\mathrm{D}}} = \varepsilon_{\parallel}' + i\varepsilon_{\parallel}''\ . \tag{8}$$

In Eq. (6) the negative root is selected because the condition $\varepsilon_{\perp}' k_{\mathrm{B,EMA}} > 0$ is required to satisfy the causality constraint [9,10]. To most clearly illustrate the broadband hyperbolic dispersion of the Ag/InGaAsP system, Supplementary Figs. 1(a) and 1(b) show the real part of the effective permittivity elements parallel and normal to $\mathbf{K}_{\mathrm{B}}$, respectively. Hyperbolic dispersion exists for all values of (λ_0,ρ) such that $\varepsilon_{\parallel}'\varepsilon_{\perp}' < 0$, which occurs throughout the plotted parameter space except for ρ<0.1. Two primary sources of losses in the system are evident from Supplementary Figs. 1(c) and 1(d), which show the imaginary part of the effective permittivity elements parallel and normal to $\mathbf{K}_{\mathrm{B}}$, respectively, with N=1x10^{16} cm^{-3}. Firstly, as the Ag fraction increases, Ohmic losses increase. Secondly, absorption at the band-edge of InGaAsP MQW leads to an abrupt increase in losses at λ_0≈1.55 µm.

To illustrate the limitations of the EMA, Supplementary Fig. 2 shows the solutions to Eq. (1) and (6) with losses omitted, for ρ=0.3, ρ=0.5, and ρ=0.7 at the pump wavelength of λ_0=1064 nm and the emission wavelength of λ_0=1550 nm. The solid (dashed) blue curves correspond to real (imaginary) parts of $\mathbf{K}_{\mathrm{B}}$, whereas the red curves correspond to the purely real $k_{\mathrm{B,EMA}}$. Optical states with transverse momentum exceeding that of the constituent MQW, $k_{\perp}/k_0$>3.5, are clearly present. As the wavelength increases from 1064 nm to 1550 nm, the EMA more closely matches the complete solution, as expected. For all wavelengths, the EMA performs best at $\mathbf{K}_{\mathrm{B}}$=0, also as expected for a local (zeroth order) theory. As the Ag ratio increases, the EMA becomes quite poor, especially at the pump wavelength. Nonetheless, the existence of non-zero real solutions to Eq. (1) shows that the Ag/InGaAsP MQW system supports hyperbolic dispersion over a large region of the (λ_0,ρ) parameter space, covering the wavelengths of the pump and all MQW emission.

Supplementary Note 2. Experimental setup and properties of control InGaAsP MQW.

A simplified schematic of the experimental setup used for characterization of the fabricated samples is shown in Supplementary Fig. 3. The half-wave plate closest to the pump (HWP 1) was first rotated to maximize the $TM_{||}$ polarized pump exiting the polarizing beam splitter (PBS). HWP 2 functioned as a variable polarization rotator. The dichroic mirror (DM) used also rotated the polarization state of the pump by a fixed 90°, such that the polarization state incident on the sample was normal to that exiting the HWP 2. While the DM reflected orthogonal polarization states exiting HWP 2 almost equally, the pump transmission through the DM was highly polarization dependent, differing by several orders of magnitude. Therefore our setup could not be used directly to measure pump reflection and we relied upon the measured photoluminescence (PL) signal to indicate the effective pump absorption. To measure polarization of the PL, the PL was passed through a linear polarizer (LP) before reaching the detector. For measurements of total PL, the LP was removed. To remove inconsistencies associated with changing the focal plane of the sample, all sample were focused such that the detected signal at the wavelength of 1550 nm was maximized.

The InGaAsP MQW wafer that we used showed no pump polarization anisotropy (PA), however, partially polarized emission was observed. Supplementary Fig. 5(a) shows the total PL induced by parallel and normal-polarized pumps at several pump powers. Supplementary Fig. 5(b) quantifies the pump PA, which is close to unity over the entire spectrum, indicating that the PL is independent of pump polarization. Supplementary Fig. 5(c) shows the total PL, along with the PL resolved into parallel and normal polarization components. A clear difference is observed. The degree-of-linear-polarization (DOLP) quantifies this difference in Supplementary Fig. 5(d), which shows that the emission is predominantly normal polarized.

To understand the origin of the peaks in the emission spectra, we theoretically calculated the spontaneous emission and gain spectra of a 10 nm InGaAsP QW, according to the method outlined in[7]. A valence band offset of $0.55(E_{G,B}-E_{G,W})$ was used where, $E_{G,B}$ and $E_{G,W}$ are the bandgap energies of the barrier and well materials, respectively, both depending on temperature and carrier density[7]. Supplementary Fig. 8 shows emission peaks at ~1550 nm, ~1450 nm, and ~1350 nm, which arise from transitions between the first conduction and heavy-hole, first conduction and light-hole, and second conduction and heavy-hole subbands, respectively. As the pumping strength is increased the spectra blue-shift due to filling of higher energy states. For pump powers used in our experiment, the peak at 1550 nm dominates the control MQW, suggesting that experimental carrier densities do not exceed $3x10^{18}$ cm^{-3}.

We also characterized emission from the InGaAsP MQW after etching into nanostructures, but prior to Ag deposition. Supplementary Figs. 13(a) and 13(b) show that the spectra of MQW prior to Ag deposition for parallel and normal pump polarizations are nearly identical, indicating that etching has negligible effect on the response of the material to different pump polarizations. After Ag deposition, however, the spectra show a strong dependence on pump polarization, as shown in Supplementary Figs. 13(c) and 13(d). Therefore the presence of Ag, and the consequent hyperbolic dispersion, is necessary to achieve extreme polarization anisotropy.

Supplementary Note 3. Numerical simulations

To better understand experimental results, we performed numerical finite-difference time-domain (FDTD, Lumerical®) simulations at pump and emission wavelengths. The LuHMS was modeled both as the exact (as-fabricated) nanostructure and by the EMA, shown schematically in Supplementary Figs. 4(a) and 4(c). The NS consists of a 100 nm tall and 40 nm wide InGaAsP pillar clad with a 20nm tall and 10 nm wide Ag layer, atop a 200 nm tall and 80 nm wide InGaAsP base. Both materials have a frequency dependent, complex-valued permittivity[5,6,8]. The EMA model consists of a 300 nm tall and 80 nm wide effective medium assuming Ag fraction of ρ=0.5. In both models periodic boundary conditions and perfectly matched layers are employed along the z-coordinate and x-coordinate, respectively. Pumping is simulated by a monochromatic plane wave of wavelength λ_0=1064 nm and polarization parallel or normal to the z-coordinate, which is parallel to the metacrystal Bloch vector $\mathbf{K}_B$. Results are shown in Supplementary Fig. 4(b). The calculated absorption anisotropies of 26 and 17 for the LuHMS modeled by exact nanostructure and EMA, respectively, are in excellent qualitative and good quantitative agreement with measured values of pump PA. The close agreement between NS and EMA models further validates the use of the EMA in describing the LuHMS. Emission is simulated similarly, but with a planewave source incident from the opposite direction. Results at the emission wavelength of 1350 nm are shown in Supplementary Fig. 4(d). The simulated DOLP is calculated as the transmission anisotropy and is seen to be in good qualitative agreement with experimental values.

To confirm that extreme anisotropy is an effect of hyperbolic dispersion and not a simple artefact independent of period size, we simulated pump behavior over a large range of period lengths. Supplementary Fig. 6(a) shows absorption of the parallel and normal-polarized pump for period lengths from 20 nm to 800 nm and constant Ag fraction of ρ=0.5. Anisotropy is strongest when the EMA is most valid and the Ag/InGaAsP system exhibits hyperbolic dispersion. As the period increases, the anisotropy becomes significantly smaller, confirming that the extreme anisotropy measured in our samples results from hyperbolic dispersion enabled by deeply subwavelength structuring. This is further supported through calculation of the modes of the system, according to Eq. (1). Supplementary Fig. 6(b) shows, for the pump vacuum wavelength of 1064 nm, that only a single parallel ($TM_{\|}$) polarized Bloch mode exists below a critical period length. As the period length increases, normal-polarized ($TE_{\perp}$) modes are supported, reducing the absorption anisotropy illustrated in Supplementary Fig. 6(a). Hence, deeply subwavelength periodicity, and hyperbolic dispersion in the effective medium limit, is required to observe extreme polarization anisotropy.

To increase absorption of normal-polarized pump absorption and PL emission, we designed a wavelength-scale grating based on, both, infinitely-extended multilayer and EMA models. Supplementary Fig. 6(c) shows simulated absorption of parallel and normal polarized pumps as a function of grating depth for the Ag/InGaAsP system modeled in the EMA with a grating of period Λ_G=390 nm and ρ=0.5. Pump PA decreases dramatically in the presence of a grating, consistent with our experimental observations. Similar results were found for different grating periods.

Supplementary Note 4. Directional propagation properties of LuHMS

It is well known that energy propagation in media with hyperbolic dispersion is highly directional, forming resonance cones[11,12]. The resonance cone half-angle determines the principal direction of energy propagation and, in the EMA, is defined as

$$\theta_{\mathrm{RC}} = \tan^{-1}\left(\sqrt{-\frac{\varepsilon_{\perp}}{\varepsilon_{\parallel}}}\right), \tag{9}$$

where the angle is measured relative to the metacrystal axis. The half-angle may also be directly described with the Poynting vector,

$$\theta_{\mathrm{RC,S}} = \tan^{-1}\left(\frac{S_{\perp}}{S_{\parallel}}\right) = \tan^{-1}\left(\frac{k_{\perp}}{\varepsilon_{\parallel}} / \frac{k_{\parallel}}{\varepsilon_{\perp}}\right), \tag{10}$$

where the parallel wave vector component is calculated by EMA or Bloch's theorem. On the other hand, the principle direction of the wave vector is given by

$$\theta_{\mathrm{k}} = \tan^{-1}\left(\frac{k_{\perp}}{k_{\parallel}}\right), \tag{11}$$

which is known to be counterposed to the Poynting vector in media with hyperbolic dispersion [13]. Supplementary Fig. 9(a) shows a schematic of the multilayer, defining the resonance cone angle and effective permittivity at this angle. In Supplementary Fig. 9(b) the dispersion of the imaginary effective permittivites is shown, while in Supplementary Fig. 9(c) the wavelength dependence of Eqs. (9)-(11) for our LuHMS made of Ag and InGaAsP MQW is shown. Regardless of the technique used to calculate the resonance cone angle, the angle of principal energy flow increases monotonically with wavelength, suggesting a mechanism for the observed difference in PL spectra of the LuHMS relative to the control MQW. As the angle increases, the wave is directed more normal to the metacrystal axis and therefore experiences more attenuation. Relative to the control MQW, shorter wavelengths are more likely to be detected because they propagate closer to the metacrystal axis and therefore experience less attenuation than longer wavelengths. For convenience, the dispersion of the imaginary effective permittivity elements are shown in Supplementary Fig. 9(b) from which we may quantitatively estimate the effect of directionality.

The attenuation, α, of a plane wave is directly proportional to the imaginary part of the permittivity. From an elementary model of the QW, assuming parabolic conduction and valence bands[8], we calculate an effective attenuation using the following relation

$$\alpha_{\mathrm{RC}}(\theta_{\mathrm{RC}}) = k_0 \frac{\varepsilon''_{\mathrm{RC}}(\theta_{\mathrm{RC}})}{\sqrt{\varepsilon'_{\mathrm{D}}}}, \tag{12}$$

where

$$\varepsilon''_{\mathrm{RC}}\left(\theta_{\mathrm{RC}}\right)=\sqrt{\left(\varepsilon''_{\parallel}\cos\theta_{\mathrm{RC}}\right)^{2}+\left(\varepsilon''_{\perp}\sin\theta_{\mathrm{RC}}\right)^{2}} \quad . \tag{13}$$

We find that emission at 1350 nm experiences ~35% less attenuation than emission at 1550 nm, shown in Supplementary Fig. 9(d). Thus we believe the wavelength dependence of the principal direction of energy propagation is observed as a blue-shifting of peak emission in the LuHMS relative to the control MQW for the same pumping conditions. This blue-shifting occurs independently of the inhomogeneous broadening associated with filling of electronic states according to the Pauli Exclusion Principle.

Supplementary Note 5. Coupling between high-*k* states and vacuum states without a grating

In conventional multilayer structures, a grating is necessary to efficiently couple the high-*k* states supported by the HMM to vacuum for detection[14–16]. By rotating the optical axis of the multilayer 90°, excitation and emission of high-*k* states becomes possible without the need of a grating. Supplementary Fig. 7(a) shows a schematic of the LuHMS, with wave-vector components parallel, $k_{\|}$, and normal, $k_{\perp}$, to the optical axis specified. Coupling of the pump beam at normal incidence (along the $k_{\perp}$ axis) into the LuHMS from vacuum, and coupling of normal emission from the LuHMS into vacuum, requires conservation of $k_{\|}$. Supplementary Figs. 7(b) and 7(c) present the wave-vector diagram of the LuHMS at λ_0=1064 nm λ_0=1550 nm, respectively, with losses omitted for clarity. Coupling occurs for HMM states with $k_{\perp}/k_0>k_{\mathrm{bulk}}$, such that $k_{\|}/k_0<1$. The black and green curves describe the states ($\pm k_{\|},k_{\perp}$) supported in vacuum and bulk MQW, respectively, while HMM states are described by blue and red curves, calculated by Bloch's Theorem and EMT, respectively. Supplementary Fig. 7(b) shows that a pump beam, consisting of a finite angular bandwidth centered at normal incidence, will excite a range of high-$k_{\perp}$ states in the HMM. Considering an experimental excitation half-angle of 24°, the angular bandwidth of the pump beam, in terms of wave-vector components ($\pm k_{\|},k_{\perp}$), is $(-0.41, 0.91)k_0 < \phi < (0.41, 0.91)k_0$. Looking at Supplementary Fig. 7(b), conservation of $k_{\|}$ then tells us that bulk and Bloch-HMM states in the ranges of $3.68<k_{\perp}/k_0<3.70$ and $5.46< k_{\perp}/k_0<5.48$, respectively, may be excited without use of a grating. Similarly, Supplementary Fig. 7(c) shows that emission over the same angular bandwidth, allows states in the ranges $3.42< k_{\perp}/k_0<3.45$ and $4.76< k_{\perp}/k_0<4.77$ to out-couple from the bulk and Bloch-HMM, respectively, into vacuum without a grating.

Supplementary Note 6. Tolerance of polarization anisotropy to small changes in geometric parameters.

To test the robustness of grating coupling to fabrication variability, we measured pump PA on LuHMS fabricated with slightly varying period and grating size. Supplementary Fig. 10 compares three LuHMS each fabricated with different electron-beam doses. Regardless of grating period, the pump PA is dramatically reduced when the LuHMS contains a grating. Furthermore, slight changes to the geometric parameters are seen to have no qualitative effect on anisotropy. In Supplementary Fig. 10, pump PA for the LuHMS without a grating is compared to pump PA in LuHMS with gratings of periods of 170 nm, 190 nm, 220 nm, 260 nm, 310 nm, 390 nm, and 520 nm. The trend of pump PA reduction via grating coupling remains consistent despite small changes to geometric parameters of the LuHMS.

Supplementary Note 7. Intensity enhancement of photoluminescence

To demonstrate that the additional high-*k* states provided by nanostructured Ag leads to enhanced PL, we performed additional experiments using a reverse excitation technique. Samples were fabricated according to the process described in Methods of the main text, with the exception that the InP capping layer was not removed. By omitting the HCl wet etch step from the process, the double-side polished samples remain smooth on both sides. Consequently, the MQW may be excited controllably from the substrate side, permitting a direct comparison between PL signals from the control MQW, flat Ag/InGaAsP interface, and nanostructured Ag/InGaAsP. Additionally, in some samples a 3 nm film of SiO_x was sputtered prior to Ag sputtering, to form an insulation layer meant to reduce quenching of plasmonic states. Supplementary Figs. 11(a) and 11(b) show PL spectra at an average pump power of 5 mW for the three cases in the absence and presence of SiO_x. Relative to the InGaAsP MQW and a single flat Ag/InGaAsP MQW interface, the nanostructured Ag/InGaAsP MQW system exhibits roughly 3.50x and 1.25x stronger PL intensities across the emission spectrum, quantified by the intensity enhancement of Supplementary Figs. 11(c) and 11(d). Total PL signals of flat and nanostructured Ag/InGaAsP MQW is increased by ~1.25x in the presence of SiO_x, while the PL from control InGaAsP changes negligibly, suggesting that quenching of high-*k* states is indeed reduced.

Supplementary Figs. 12(a) and 12(b) show the integrated PL as a function of average pump power for the three cases. Relative to the InGaAsP MQW and single, flat Ag/InGaAsP MQW interface, the nanostructured Ag/InGaAsP MQW system exhibits ~3.0x-3.5x and 1.1x-1.3x stronger integrated PL across the range of pump powers studied, quantified by the intensity enhancement of Supplementary Figs. 12(c) and 12(d). Further, we observe that the integrated PL signals of flat and nanostructured Ag/InGaAsP MQW is increased by ~1.25x in the presence of SiO_x, while the PL from control InGaAsP changes negligibly, again suggesting that quenching of high-*k* states is reduced.

Supplementary Note 8. Principle and realization of meta-gain media.

The observed behavior of the LuHMS may be generalized by considering the main "ingredients" of the fabricated system in abstract terms. Essentially, we have demonstrated that the simultaneous co-optimization of pump properties, electronic density of states (DOS), and optical DOS leads to engineered meta-gain spectra with properties beyond those of the constituent gain media. We show this schematically in Supplementary Fig. 14(a). Pump properties include, but are not limited to, polarization and power. Electronic DOS may be continuous, step-like, inverse square root like, or delta-function like, representative of bulk, quantum well, quantum wire, and quantum dot semiconductors, respectively[1]. Optical DOS may be elliptical, closed hyperbolic, or open hyperbolic, representative of conventional dielectric, and type I and type II hyperbolic metamaterials, respectively[2].

Abstracting the LuHMS based on Ag/InGaAsP MQW in terms of these parameters, we see in Supplementary Figs. 14(b), 14(c), and 14(d) that different combinations of pump polarization and optical DOS, with a fixed electronic DOS, leads to significantly different PL spectra and evolution with pump power. Thus the gain spectra seen by a weak probe signal is expected to be engineered with properties unobtainable with the MQW alone.

Supplementary References

1. Thé, G. A. P. How to simulate a semiconductor quantum dot laser: general description. *Rev. Bras. Ensino Física* **31,** 23021–23028

2. Cortes, C., Newman, W., Molesky, S. & Jacob, Z. Quantum nanophotonics using hyperbolic metamaterials. *J. Opt.* **14,** 63001 (2012).

3. Rytov, S. M. Electromagnetic properties of a finely stratified medium. *J. Exp. Theor. Phys.* **2,** 466 (1956).

4. Yariv, A. & Yeh, P. in *Photonics: Optical electronics in modern communications* 539–601 (Oxford University, 2007).

5. Johnson, P. & Christy, R. Optical constants of noble metals. *Phys Rev B* **6,** 4370–4379 (1972).

6. Humphreys, D. A., King, R. J., Jenkins, D. & Moseley, A. J. Measurement of absorption coefficients of Ga0.47In0.53As over the wavelength range 1.0–1.7 μm. *Electron. Lett.* **21,** 1187–1189 (1985).

7. Coldren, L., Corzine, S. & Masanovic, M. in *Diode lasers and photonic integrated circuits* 157–246 (Wiley, 2012).

8. Smalley, J., Gu, Q. & Fainman, Y. Temperature dependence of the spontaneous emission factor in subwavelength semiconductor lasers. *IEEE J Quant Elect* **50,** 175–185 (2014).

9. Podolskiy, V. in *Tutorials in Metamaterials* (eds. Noginov, M. & Podolskiy, V.) 163–207 (CRC Press, 2012).

10. Smalley, J., Vallini, F., Kante, B. & Fainman, Y. Modal amplification in active waveguides with hyperbolic dispersion at telecommunication frequencies. *Opt. Exp.* **22,** 21088–21105 (2014).

11. Fisher, R. K. & Gould, R. W. Resonance Cones in the Field Pattern of a Short Antenna in an Anisotropic Plasma. *Phys. Rev. Lett.* **22,** 1093–1095 (1969).

12. Newman, W. D., Cortes, C. L. & Jacob, Z. Enhanced and directional single-photon emission in hyperbolic metamaterials. *J. Opt. Soc. Am. B* **30,** 766–775 (2013).

13. Mackay, T. & Lakhtakia, A. in *Electromagnetic Anisotropy and Bianisotropy: a Field Guide* 106–118 (2009).

14. Lu, D., Kan, J., Fullerton, E. & Liu, Z. Enhancing spontaneous emission rates of molecules using nanopatterned multilayer hyperbolic metamaterials. *Nat. Nanotechnol.* **9,** 48–53 (2014).

15. Sreekanth, K., Krishna, K., De Luca, A. & Strangi, G. Large spontaneous emission rate enhancement in grating coupled hyperbolic metamaterials. *Sci. Rep.* **4,** 6340 (2014).

16. Galfsky, T. *et al.* Active hyperbolic metamaterials: enhanced spontaneous emission and light extraction. *Optica* **2,** 62–65 (2015).
